# Preparation and in-situ Raman characterization of binder-free u-GF@CFC cathode for rechargeable aluminum-ion battery

**DOI:** 10.1016/j.mex.2019.10.008

**Published:** 2019-10-14

**Authors:** Chengyuan Liu, Zhiwei Liu, Hongkun Niu, Cong Wang, Zhaowen Wang, Bingliang Gao, Jingjing Liu, Mark Taylor

**Affiliations:** a School of Metallurgy, Northeastern University, Shenyang, 110819, China; b Department of Chemical and Materials Engineering, University of Auckland, Auckland, 1142, New Zealand

**Keywords:** Preparation  and in-situ Raman characterization of binder-free u-GF@CFC cathode for rechargeable aluminum-ion battery, Aluminum-ion battery, Binder-free, u-GF@CFC cathode, In-situ Raman

## Abstract

We provide a method for preparation of binder-free cathode for rechargeable aluminum-ion batteries (AIBs). Ultrasonicated natural graphite (u-NG) flakes in N-methylpyrrolidone (NMP) is drop-casted to a carbon fiber cloth (CFC) to obtain binder-free u-GF@CFC cathode for AIBs. We also provide an in-situ Raman spectral technology and the corresponding in-situ Raman cell to determine the mechanism of intercalation/deintercalation reactions of the chloroaluminate ions at cathode of AIBs. The in-situ Raman spectra are recorded using a Raman spectrometer combined with a potentiostat/galvanostat model electrochemical workstation.

• A simple preparation method of a binder-free u-GF@CFC cathode is suggested.
• The u-GF@CFC cathode is obtained by drop-casting ultrasonicated graphite flakes on the surface and internal gaps of the carbon fiber cloth.
• The method includes the in-situ Raman spectral technology and the corresponding in-situ Raman cell.

## Graphical abstract

**Figure:**
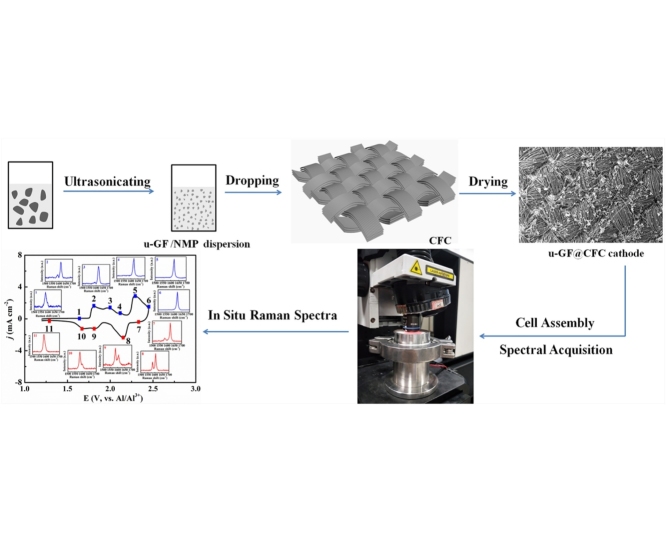

**Specification Table**

**Table:** 

Subject Area:	Energy
More specific subject area:	Aluminum-ion battery, in-situ spectral technology
Method name:	Preparation and in-situ Raman characterization of binder-free u-GF@CFC cathode for rechargeable aluminum-ion battery
Name and reference of original method:	1. K.V. Kravchyk, S. Wang, L. Piveteau, M.V. Kovalenko, Efficient Aluminum Chloride-Natural Graphite Battery, Chem. Mater., 29(2017), pp. 4484-4492; 2. A.S. Childress, P. Parajuli, J.Y. Zhu, R. Podila, A.M. Rao, A Raman spectroscopic study of graphene cathodes in high-performance aluminum ion batteries, Nano Energy, 39(2017), pp. 69-76
Resource availability:	graphite flakes, natural (99.9%, Alfa Aesar); carbon fiber cloth (CeTech Co. Ltd. Taiwan)

## Method details

### Overview

Aluminum-ion batteries (AIBs) are considered as one of new electric energy storages on a large-scale because aluminum has an excellent theoretical volumetric capacity (8040 mA h cm^−3^) which is four times higher than that of lithium (2046 mA h cm^−3^). Additionally, aluminum is the most abundant metal element in the earth crust and can exchange three electrons during electrochemical process [1,2]. Carbon-based materials are considered as the most promising cathode materials for AIBs because carbon-based cathodes exhibit higher discharge voltage platform than other materials (such as oxides and sulfide) and deliver suitable specific capacity and energy density [2,3]. Dai and co-workers reported an aluminum-ion battery with SP-1 natural graphite flakes (NG) and a polyvinylidene fluoride (PVDF) binder as the cathode. The Al-graphite battery delivers a specific capacity of 110 mA h g^-1^ with a high coulombic efficiency and good cycle stability [4]. However, commercial graphite materials easily expand due to the intercalation/de-intercalation reaction of chloroaluminate ions, resulting in pulverization of cathode and short cycle life of the battery. In contrast, the pyrolytic graphite (PG) foil does not expand obviously during charging/discharging and has a comparable specific capacity to NG [5]. Graphene/graphitic foam prepared by the chemical vapor deposition (CVD) at near 1000 ℃ can provide excellent storage space for intercalation/de-intercalation reactions of chloroaluminate ions due to the three-dimensional structures. The aluminum-graphene/graphitic foam batteries exhibit high specific capacity of ∼120 mA h g^-1^ at a current density of 5000 mA g^-1^ and can stably operate for thousands of cycles [[5], [6], [7]]. Chen et al. prepared a reduced graphene oxide (rGO) aerogel cathode with highly crystallized defect-free few-layer graphene lattice by chemical reduction of graphene oxide (GO) using N_2_H_4_ vapor, followed by thermal annealing at 3000 ℃. The cathode affords a high specific capacity of 100 mA h g^-1^ at 5000 mA g^-1^ and an excellent cycle stability in 25,000 cycles [8]. Subsequently, they reported a “trihigh tricontinuous” (3H3C) graphene film cathode for AIBs. The graphene film is prepared in following steps: firstly, GO film is obtained by a classical wet-spinning method; secondly, by chemical reduction in N_2_H_4_ vapor to obtain rGO film; and finally annealed at 2850 ℃ to obtain final cathode material. The cathode retains high specific capacity of around 120 mA h g^-1^ at ultrahigh current density of 400 A g^-1^ after 250000 cycles [9]. Although the high-quality graphitic foam/graphene cathodes exhibit a high specific capacity, high preparation temperature (about 1000 ℃, even higher than 2500 ℃) and high energy consumption hinder its large-scale production and commercial applications. Wang et al. fabricated graphene nanosheet (GN) paper cathode for AIBs. The GN paper is prepared in following steps: pristine graphite powders are stirred in an ethyl alcohol aqueous solution; followed by further exfoliated in H_2_SO_4_/HNO_3_ solution; after pH adjustment to 7, the clean suspension is transferred onto the filter membrane under the vacuum to create the GN paper [10]. Smajic reported a cathode using rGO as active material and PVDF as binder. rGO is prepared via hydrothermal reduction of GO powders, then dried in supercritical CO_2_ [11]. The GN and rGO used as the active materials afford a high specific capacity of >100 mA h g^-1^at a current density of 1000 mA g^-1^.

At present, it is a common method to make film cathode by combining active material with a binder. Wang and Uemura evaluated that the binders are incompatible with acidic AlCl_3_-based electrolyte and seriously damaged resulting in a low discharge capacity of AIBs [12,13]. Kovalenko proved that flaky graphite particles after ultrasonication are more suitable for the cathode of AIBs than spheroidized particles [14,15]. Recently, we proposed a simple method to prepare a binder-free composite cathode used for AIBs by drop-casting small ultrasonicated graphite flakes (u-GF) on carbon fiber cloth (CFC) [16].

Spectroelectrochemistry is often used to elucidate reactions of electrode. In-situ Raman spectroscopy is applied to many fields [[17], [18], [19], [20], [21]], preferentially used to characterize the reaction mechanism on graphite electrode due to that the G-band at ∼1585 cm^−1^ in Raman spectra of graphite is highly sensitive to the structural changes during interlayer interactions [[22], [23], [24], [25], [26]]. The reaction on the cathode of aluminum-graphite battery is a typical behavior of graphite interlayer compounds (GIC), that is the intercalation/deintercalation of chloroaluminate ions into graphite layers, so Raman spectroscopy is widely used to determine the reaction mechanism of AIBs [4,5,8,[27], [28], [29]]. Reliable sealing performance of Raman cell and suitable experimental parameters are required for Raman spectral acquisition because AlCl_3_-based electrolyte may be destroyed by reacting with moisture or oxygen, and graphite is easily damaged by the laser at high power. However, the information about the assembly of Raman cell and signal acquisition of Raman spectra is ambiguous or incomplete in previous studies [4,5,8,[27], [28], [29]]. In this method, we design an in-situ Raman cell and provide the detailed spectral acquisition technology for carbon-based cathode of AIBs.

### Preparation of u-GF@CFC cathode

**Fig. 1:**
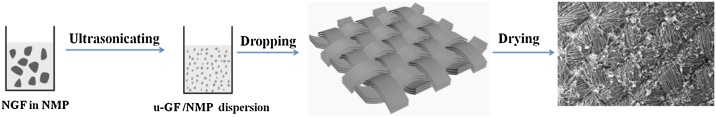
A schematic representation of the preparation process for u-GF@CFC cathode.

A binder-free and three-dimensional cathode for aluminum-ion battery is prepared through ultrasonication and drop-casting steps, as depicted in Fig. 1. 0.2 g of natural graphite flakes (NGF, ∼2000 μm, 99.9%, Alfa Aesar) were placed in a glass vial, and 6 mL anhydrous N-methylpyrrolidone (NMP, 98%, Aladdin) was added, then the NGF-NMP mixture was ultrasonicated using a ultrasonic homogenizer (240 W, 40 kHz). After ultrasonication for hours, a part of large graphite flakes rested at the bottom of the bottle, the rest dispersed into the NMP solution which was named as ultrasonicated graphite flakes-NMP (u-GF/NMP) dispersion and was used for making cathode of AIBs. The dispersion was then drop-casted to a carbon fiber cloth (CFC, 125 g m^−2^, CeTech Co. Ltd. Taiwan), followed by drying in a vacuum oven at 80 ℃ for 12 h to obtain the ultrasonicated graphite flakes@carbon fiber cloth (u-GF@CFC) cathode.

### In-situ Raman cell and Raman spectra characterization

**Fig. 2:**
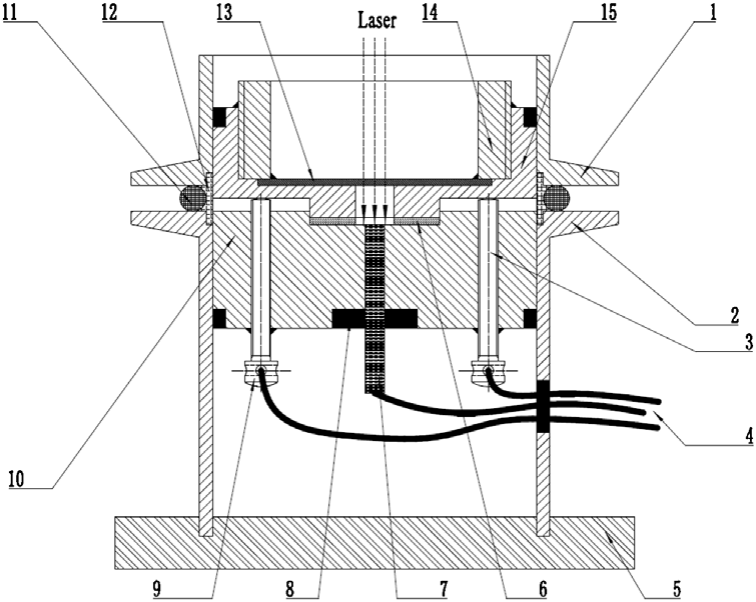
Three-electrode in-situ Raman cell at the fully assembled stage.

**Fig. 3:**
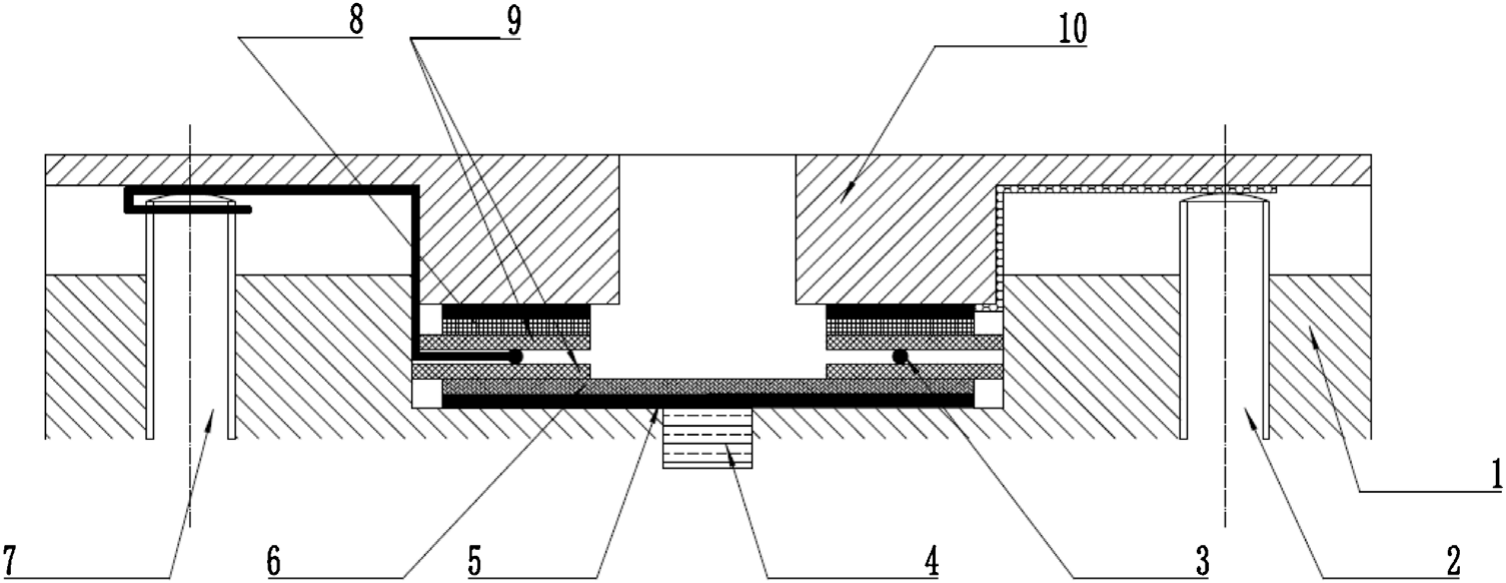
The structural details of three-electrode assembly in the in-situ Raman cell.

A conventional AIB is composed of carbon-based material as cathode, metal aluminum as anode, and aluminum chloride-based ionic liquid as electrolyte. Aluminum chloride-based electrolyte is sensitive to water and oxygen, and highly corrosive to many metals [30], hence a Raman cell used for AIBs must have good corrosion resistance and good sealing. We design and assemble a Raman cell used for in-situ characterizing the carbon-based cathode for AIBs during charging/discharging. The structure of in-situ Raman cell is shown in Fig. 2:

1) The upper/lower cavities (10, 15) are made of polytetrafluoroethylene material;
2) The above cavities are fitted into a set of KF 50 flanges (1, 2, 11, 12);
3) A quartz wafer (13) of 1 mm thickness is placed in the upper cavity and fixed by a set collar (14). The distance between the WE and the quartz plate is 7 mm, which is ensured to be smaller than the focal length of the objective of the Raman spectroscopy;
4) A tungsten bar (7), two stainless steel bars (3, 9) are used as electrode current collectors, respectively;
5) The wires (4) are used for connecting to an electrochemical workstation;
6) The contact points of all components are filled with sealant (8) to improve the leakproofness of the device;
7) A support pedestal (5) makes the device more stable.
8) The battery body (6) is mainly consisted of three electrodes and separator. Its structural details are shown in Fig. 3.

1-KF 50-20 flange; 2-KF 50-50 flange; 3-contact pole for counter electrode; 4-three electrode connecting wires; 5-support pedestal; 6-battery body; 7- contact pole for working electrode; 8-sealant; 9-contact pole for reference electrode; 10-lower cavity; 11-seal ring; 12-seal stent; 13-quartz wafer; 14-set collar; 15-upper cavity

In-situ Raman cell is assembled in a glove box with the contents of moisture and oxygen less than 0.1 ppm (MBRAUN MB 200B, Germany):

1) The working electrode (WE), carbon-based material (Φ16 mm) under investigation, is contacted with tungsten bar by binding with carbon tape. An aluminum foil (Φ16 mm) as counter electrode (CE) is fixed on the upper cavity by binding with carbon tape;
2) Then the reference electrode (RE), an aluminum wire (Φ0.5 mm) with a ring-shaped front (Φ12 mm), is fixed between two layers of glass fiber filter papers (Φ20 mm) which are as separators. They are placed on the upper surface of the WE;
3) Small holes (Φ6-8 mm) in the center of the Al foil and separators enable the exciting laser beam focus onto the WE;
4) A suitable amount of electrolyte (AlCl_3_-based ionic liquid) is added to ensure the WE and glass fiber filter papers are completely saturated with the electrolyte;
5) Finally, the cell is assembled and taken out of the glove box for in situ Raman spectra measurement.

1-lower cavity; 2-counter electrode connection pole; 3-reference electrode; 4-working electrode connection pole; 5-carbon tape; 6-working electrode; 7-reference electrode connection pole; 8-counter electrode; 9-separator; 10-upper cavity

**Fig. 4:**
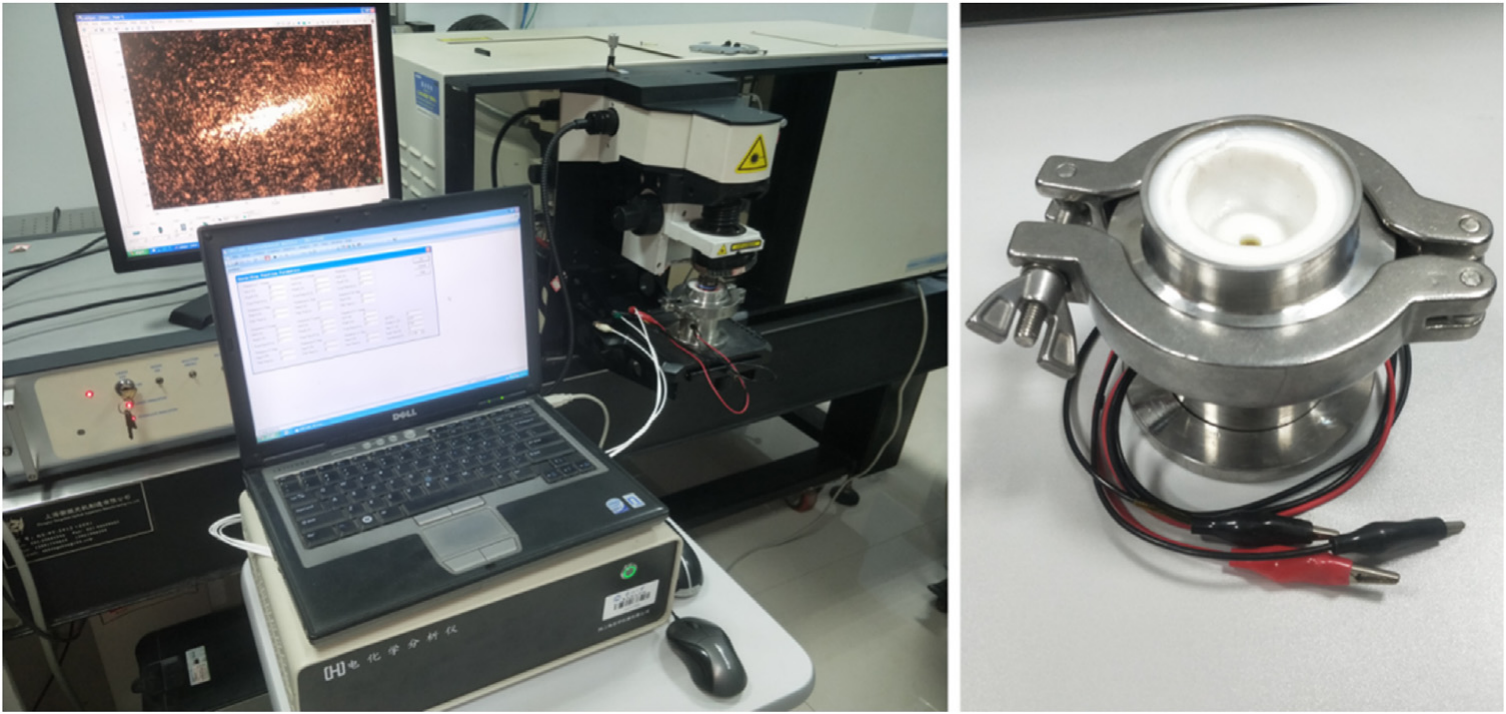
Photos of in-situ Raman measurement system used for AIBs.

The in-situ Raman spectra are recorded using a Raman spectrometer combined with a potentiostat/galvanostat model electrochemical workstation (Fig. 4). Cyclic voltammetry (CV) and sweep step function (SSF) measurements are performed using a potentiostat/galvanostatmodel CHI 660E (CH Instruments). A cyclic voltammetry curve is recorded to determine the Raman signal acquisition points. Then, electrochemical signals are applied to the electrodes by the sweep step function (SSF) technology, meanwhile the Raman spectra are recorded using a HR 800 Raman spectrometer (Horiba Jobin Yvon LabRAM). The excitation light of an He-Ne laser at 632.8 nm wavelength is focused on the WE surface through a 50× objective (Olympus) with a focal length of 10 mm. The confocal slit is adjusted to be minimum to avoid the effect of non-confocal signal. The scattered light is collected in a backscattering geometry along the same optical path as the pumping laser. The power of laser beam delivered to the electrode surface is roughly 1.2 mW to avoid damaging to the cathode. The spectrum acquisition time is set as 15 s with 2 accumulations.

## Method validation

**Fig. 5:**
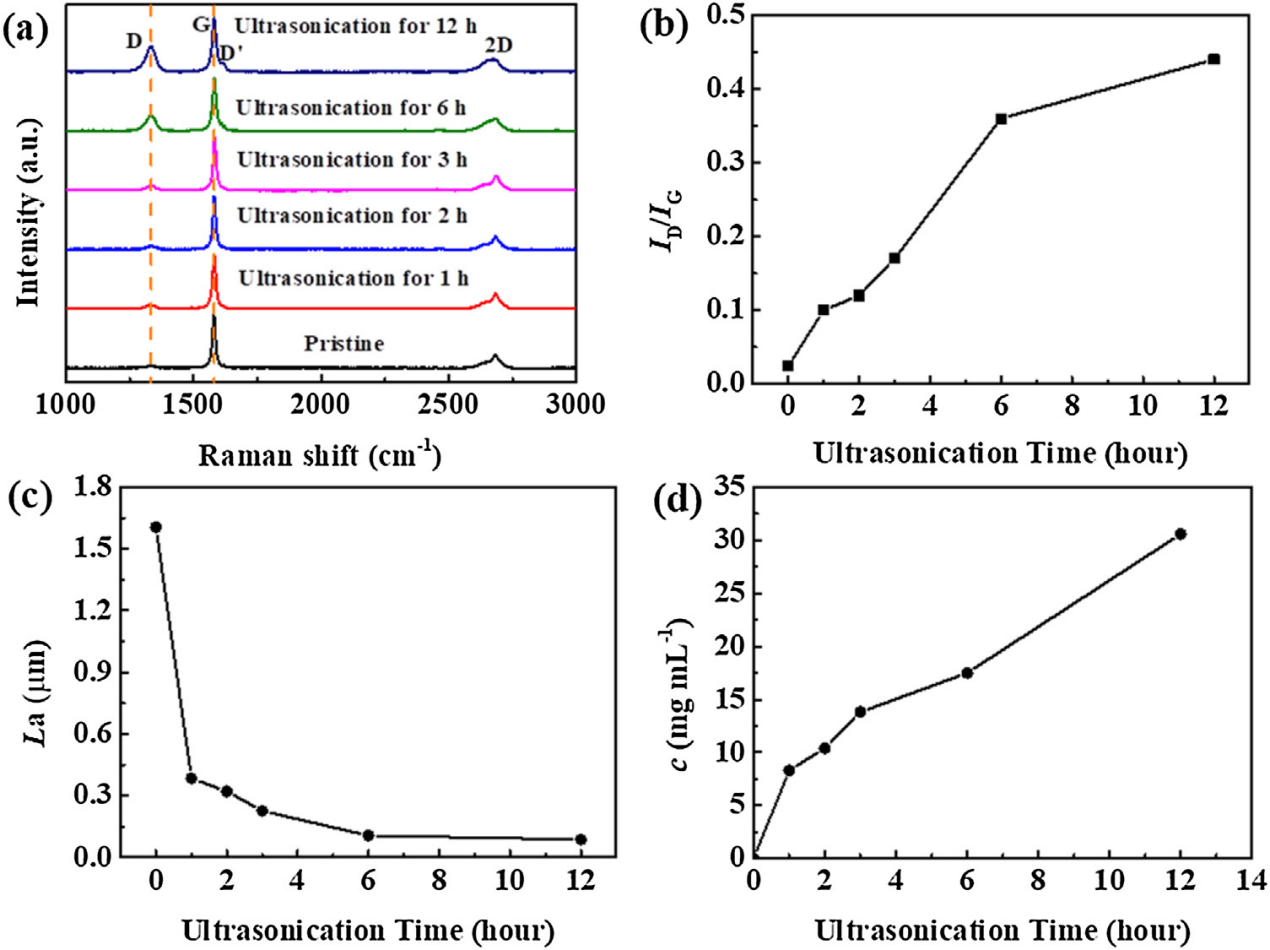
(a) Raman spectra of the graphite flakes before and after ultrasonication in NMP; variation of *I*_D_/*I*_G_ (b) and *L*a (c) of u-GF obtained with various ultrasonication time; (d) variation of the concentration of u-GF in dispersion after various ultrasonication time.

To characterize the properties of u-GF, the u-GF/NMP dispersion was centrifuged at 2500 rpm for 20 min. The collected precipitate was dried under vacuum at 80 ℃ for 12 h to obtain ultrasonicated graphite flakes. U-GF obtained with various ultrasonication time were referred to as u-GF (1 h), u-GF (2 h), u-GF (3 h), u-GF (6 h), u-GF (12 h). Several researchers [4,8,9] have confirmed that defects in graphitic cathode are harmful for the performance of AIBs, so the effect of ultrasonication time on defects in u-GF was investigated. Fig. 5(a) shows the Raman spectra of the graphite flakes before and after ultrasonication in NMP. The D peak represents the defects in graphite. *I*_D_/*I*_G_ was calculated to estimate the degree of defects in the materials [30]. The values of *I*_D_/*I*_G_ are shown in Fig. 5(b), it indicates that the number of defects in u-GF increase with increasing of ultrasonication time. The 2D peak (2700 cm^−1^) is almost as same as pristine graphite which means that u-GF with ultrasonication time less than 3 h still maintains the typical graphite structure. In contrast, the 2D peak and D peak of u-GF (6 h) and u-GF (12 h) show obvious difference from pristine graphite, which indicates that they may have more defects than the pristine graphite. Besides, a new shoulder peak D' occurred at 1614 cm^-1^ reveals that new defects are formed in u-GF (6 h) and u-GF (12 h). The average size of the graphitic crystallite (*L*a) can be calculated using the Eq. (1) [31] and the values of *L*a of u-GF are shown in Fig. 5(c). *L*a decreases with increasing of ultrasonication time.

(1) $$L\text{a}\left(\text{nm}\right)=\left(2.4\times 10^{-10}\right)\lambda_{\text{laser}}^{4}{\left(I_{\text{D}}/I_{\text{G}}\right)}^{-1}$$

**Fig. 6:**
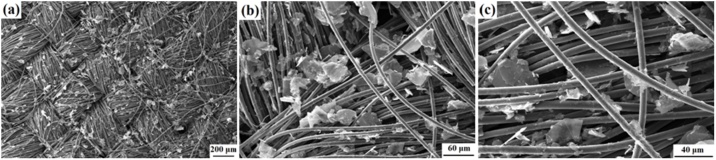
(a–c) SEM images of u-GF (3 h)@CFC.

Where λ_laser_ is the wavelength of the laser source (i.e. 633 nm). Although the concentration of u-GF in dispersion increases with increasing of ultrasonication time increased (Fig. 5(d)), ultrasonication time has a negative effect on the defects in u-GF. Therefore, it is worth considering the impact of ultrasonication time on properties of u-GF used for cathode material of AIBs. Fig. 6 shows the SEM images of a binder-free cathode prepared by drop-casting u-GF (3 h)/NMP dispersion to a CFC with three-dimensional structure. Small graphite flakes distribute evenly on the out surface and internal gaps of the carbon fiber cloth.

**Fig. 7:**
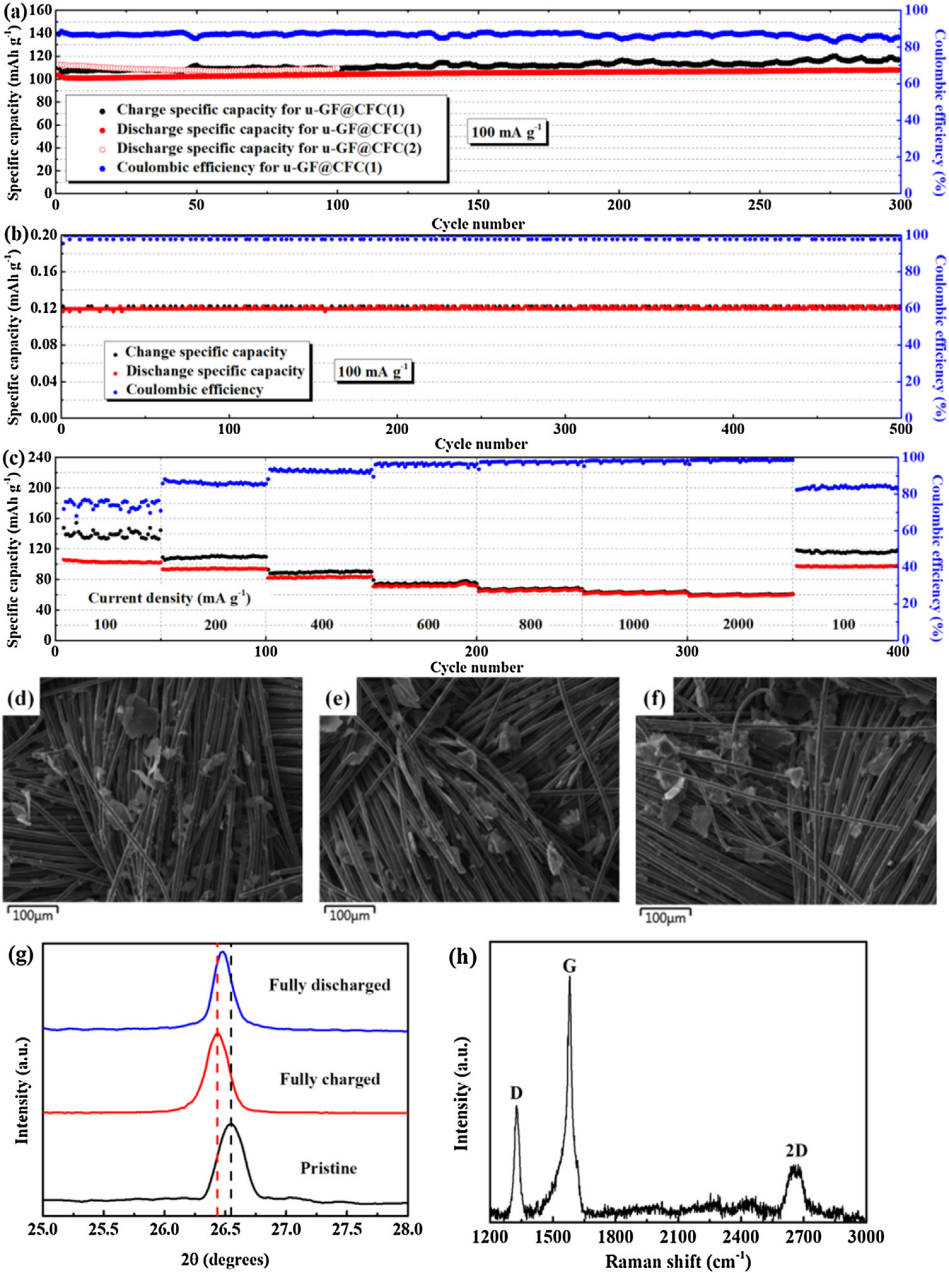
The cycling performances of aluminum/u-GF@CFC battery (a) and aluminum/CFC battery (b) at 100 mA g^−1^; (c) rate capability of Al/u-GF@CFC battery at current densities ranging from 100 to 2000 mA g^−1^; SEM images of u-GF@CFC at pristine (d), fully charged (e) and fully discharged (f) state; (g) XRD patterns (g) and Raman spectrum (h) of u-GF in aluminum/u-GF@CFC battery after charge/discharge cycling.

The novel u-GF@CFC cathode has been successfully used for aluminum-ion battery and assembled in Al|EMImCl-1.3AlCl_3_|u-GF@CFC batteries, which has excellent cycling stability and performance [16]. The u-GF@CFC cathode delivers a high discharge specific capacity of >100 mA h g^−1^ at 100 mA g^−1^ (Fig. 7(a)), and the battery can operate over 300 cycles stably. The negligible specific capacity of CFC (∼0.12 mA h g^-1^ at 100 mA g^-1^) indicates that the u-GF is the active material and contribute all the capacity of Al/u-GF@CFC battery (Fig. 7(b)). We also prepared another cathode through the same method (referred as u-GF@CFC(2)) and tested its electrochemical performance. The comparable discharge specific capacity of u-GF@CFC (2) confirms the reproducibility of cathode. In addition, u-GF@CFC cathode exhibits outstanding rate capability (Fig. 7(c)), especially the cathode displays high discharge specific capacity of about 60 mA h g^−1^ at 1000 and 2000 mA g^−1^. Series of characterizations are conducted to probe the structural evolution of u-GF@CFC cathode after 300 cycles. Remarkably, it is found that the 3D structure of u-GF@CFC is undestroyed and u-GF is well adsorbed on the CFC after long-term cycle from the SEM images of u-GF@CFC (Fig. 7(d–f)). A typical (002) diffraction peak is observed from the XRD patterns of u-GF with different state (Fig. 7(g)), which means the u-GF remains the typical structure of graphite. The interplanar distance increases from 3.356 to 3.376 Å when the battery is fully charged and then restores to a smaller value after it fully discharged, which is attributed by the intercalation/deintercalation reaction of AlCl_4_- into graphite layers. Besides, Raman spectrum of the u-GF basically maintains its original structure (Fig. 7(h)) after the long-term cycling. The above analyses indicate that u-GF@CFC cathode prepared by above method has good stability and recuperability which contribute to long-term cycling life and high rate performance of the battery.

**Fig. 8:**
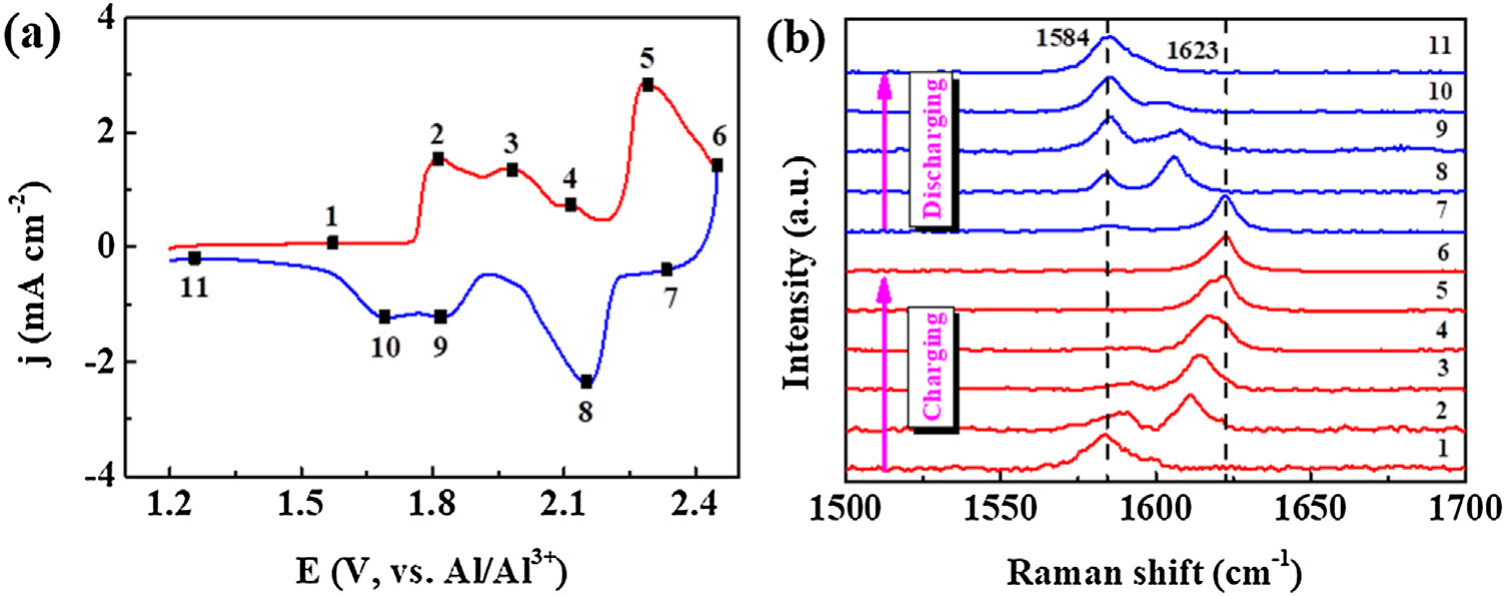
(a) Cyclic voltammetry curve of an Al/u-GF@CFC battery; (b) in-situ Raman spectra recorded for the u-GF@CFC cathode.

The in-situ Raman spectra of Al/u-GF@CFC battery with aluminum chloride-1-ethyl-3-methylimidazolium chloride (AlCl_3_-EMImCl, molar ratio = 1.3) ionic liquid as electrolyte were recorded via above method. The Raman signal acquisition points were determined by the cyclic voltammetry curve, as seen in Fig. 8(a). Several obvious anodic peaks (points 2, 3, 4, 5) and reduction peaks (points 7, 8, 9, 10) are correlated to different intercalation/deintercalation stages of chloroaluminate ions into graphite layers. The voltages, marked as points 1, 6, 11, were selected to acquire the Raman spectra of cathode at pristine, fully charged and fully discharged states, respectively. Electrochemical signals were applied to the electrodes by the SSF technology, and the parameters for a charging process were shown in Table 1. The potentiostatic steps were adopted to ensure a complete reaction of the cathode with chloroaluminate ions, meanwhile the Raman spectra were recorded using Raman spectrometer.

**Table 1:** The parameter details for a charging process of in-situ Raman cell.

Sequence	Parameters	
Step 1	Init E = 1.2 V, Final E = 1.6 V, Scan rate = 1 mV s^−1^	
Step 2	Step E = 1.6 V, Time = 600 s	Point 1
Step 3	Init E = 1.6 V, Final E = 1.85 V, Scan rate = 1 mV s^−1^	
Step 4	Step E = 1.85 V, Time = 600 s	Point 2
Step 5	Init E = 1.85 V, Final E = 2 V, Scan rate = 1 mV s^−1^	
Step 6	Step E = 2 V, Time = 600 s	Point 3
Step 7	Init E = 2 V, Final E = 2.15 V, Scan rate = 1 mV s^−1^	
Step 8	Step E = 2.15 V, Time = 600 s	Point 4
Step 9	Init E = 2.15 V, Final E = 2.3 V, Scan rate = 1 mV s^−1^	
Step 10	Step E = 2.3 V, Time = 600 s	Point 5
Step 11	Init E = 2.3 V, Final E = 2.45 V, Scan rate = 1 mV s^−1^	
Step 12	Step E = 2.45 V, Time = 600 s	Point 6

The in-situ Raman spectra recorded at various voltages were also shown in Fig. 8(b). As the charge voltage increases, G-band at 1854 cm^−1^ is split into two Raman modes of a lower-frequency component E_2g2i_ (1588 cm^-1^) for vibrations of carbon atoms in un-intercalated graphite layer and a higher-frequency component E_2g2b_ (1611 cm^-1^) for vibrations of carbon atoms in intercalated graphite layer, respectively. The peaks are right-shifted and the intensity of E_2g2i_ peak decreases with increasing of charging voltage because more intercalated graphite layers are formed due to the intercalation of AlCl_4_- into unperturbed graphite layers, then the E_2g2i_ peak disappears and a new sharp peak appears at 1623 cm^-1^ at a fully charged state, which means that the unperturbed graphene layers are intercalated by AlCl_4_- completely [16]. The reversed spectral changes are observed in the discharge process. The reversible intercalation/de-intercalation reactions in the cathode indicate a graphite intercalation compounds (GIC) type behavior [4,5,8,16,[27], [28], [29]]. According to the literature [29], the peak positions of G-band are as a function of reciprocal stage index for acceptor type GICs. Therefore, the new peak at 1623 cm^-1^ is confirmed to be a stage 2-1 GIC.

## Conclusion

The effect of ultrasonication time on the properties of ultrasonicated graphite flakes (u-GF) were studied. It can be concluded that ultrasonication time influences the number of defects in u-GF, average size of the graphitic crystallite (*L*a) of u-GF and concentration of u-GF/NMP dispersion. The u-GF/NMP dispersion obtained by ultrasonication for less than 6 h is more suitable for preparing the cathode of AIBs. A binder-free u-GF@CFC cathode is prepared via a simple method that the u-GF are evenly adsorbed on the out surface and internal gaps of the carbon fiber cloth (CFC) by drop-casting u-GF/NMP dispersion to CFC. A self-designed in-situ Raman cell and the detailed information about in-situ Raman signal acquisition technology are described. A graphite intercalation compounds (GIC) type behavior is confirmed by the reversible intercalation/de-intercalation reactions of chloroaluminate ions into the u-GF via the in-situ Raman spectra of Al/u-GF@CFC battery.

## References

[1] Elia G.A., Marquardt K., Hoeppner K., Fantini S., Lin R., Knipping E., Peters W., Drillet J.F., Passerini S., Hahn R. (2016). An overview and future perspectives of aluminum batteries. Adv. Mater..

[2] Zhang Y., Liu S.Q., Ji Y.J., Ma J.M., Yu H.J. (2018). Emerging nonaqueous aluminum-ion batteries: challenges, status, and perspectives. Adv. Mater..

[3] Das S.K. (2018). Graphene: a cathode material of choice for aluminum-ion batteries. Angew. Chem. Int. Ed..

[4] Wang D.Y., Wei C.Y., Lin M.C., Pan C.J., Chou H.L., Chen H.A., Gong M., Wu Y.P., Yuan C.Z., Angell M., Hsieh Y.J., Chen Y.H., Wen C.Y., Chen C.W., Hwang B.J., Chen C.C., Dai H.J. (2017). Advanced rechargeable aluminium ion battery with a high-quality natural graphite cathode. Nat. Commun..

[5] Lin M.C., Gong M., Lu B.A., Wu Y.P., Wang D.Y., Guan M.Y., Angell M., Chen C.X., Yang J., Hwang B.J., Dai H.J. (2015). An ultrafast rechargeable aluminium-ion battery. Nature.

[6] Yu X.Z., Wang B., Gong D.C., Xu Z., Lu B.A. (2017). Graphene nanoribbons on highly porous 3D graphene for high-capacity and ultrastable Al-ion batteries. Adv. Mater..

[7] Zhang Q.F., Wang L.L., Wang J., Xing C.Y., Ge J.M., Fan L., Liu Z.M., Lu X.L., Wu M.G., Zhang H., Lu B.A. (2018). Low-temperature synthesis of edge-rich graphene paper for high-performance aluminum batteries. Energy Storage Mater..

[8] Chen H., Guo F., Liu Y.J., Huang T.Q., Zheng B.N., Ananth N., Xu Z., Gao W.W., Gao C. (2017). A defect-free principle for advanced graphene cathode of aluminum-ion battery. Adv. Mater..

[9] Chen H., Xu H.Y., Wang S.Y., Huang T.Q., Bin X.J., Cai S.Y., Guo F., Xu Z., Gao W.W., Gao C. (2017). Ultrafast all-climate aluminum-graphene battery with quarter-million cycle life. Sci. Adv..

[10] Wang P., Chen H.S., Li N., Zhang X.Y., Jiao S.Q., Song W.L., Fang D.N. (2018). Dense graphene papers: toward stable and recoverable Al-ion battery cathodes with high volumetric and areal energy and power density. Energy Storage Mater..

[11] Smajic J., Alazmi A., Batra N., Palanisamy T., Anjum D.H., Costa P.M.F.J. (2018). Mesoporous reduced graphene oxide as a high capacity cathode for aluminum batteries. Small.

[12] Wang H.L., Bai Y., Chen S., Luo X.Y., Wu C., Wu F., Lu J., Amine K. (2015). Binder-free V_2_O_5_ cathode for greener rechargeable aluminum battery. ACS Appl. Mater. Interface.

[13] Uemura Y., Chen C.Y., Hashimoto Y., Tsuda T., Matsumoto H. (2018). Graphene nanoplatelet composite cathode for a chloroaluminate ionic liquid-based aluminum secondary battery. ACS Appl. Energy Mater..

[14] Kravchyk K.V., Wang S., Piveteau L., Kovalenko M.V. (2017). Efficient aluminum chloride-natural graphite battery. Chem. Mater..

[15] Wang S.T., Kravchyk K.V., Krumeich F., Kovalenko M.V. (2017). Kish graphite flakes as a cathode material for an aluminum chloride-graphite battery. ACS Appl. Mater. Interface.

[16] Liu C.Y., Liu Z.W., Li Q.M., Niu H.K., Wang C., Wang Z.W., Gao B.L. (2019). Binder-free ultrasonicated graphite flakes@carbon fiber cloth cathode for rechargeable aluminum-ion battery. J. Power Sources.

[17] Qiao Y., Wu S.C., Yi J., Sun Y., Guo S.H., Yang S.X., He P., Zhou H.S. (2017). From O_2_^−^ to HO_2_^−^: reducing by-products and overpotential in Li-O_2_ batteries by water addition. Angew. Chem. Int. Ed..

[18] Zhu H.L., Yu G.S., Guo Q.H., Wang X.J. (2017). In situ Raman spectroscopy study on catalytic pyrolysis of a bituminous coal. Energy Fuel.

[19] Dong J.C., Zhang X.G., Martos V.B., Jin X., Yang J., Chen S., Yang Z.L., Wu D.Y., Feliu J.M., Williams C.T., Tian Z.Q., Li J.F. (2019). In situ Raman spectroscopic evidence for oxygen reduction reaction intermediates at platinum single-crystal surfaces. Nat. Energy.

[20] Cyriac J., Wleklinski M., Li G.T., Gao L., Cooks R.G. (2012). In situ Raman spectroscopy of surfaces modified by ion soft landing. Analyst.

[21] Qiao Y., Guo S.H., Zhu K., Liu P., Li X., Jiang K.Z., Sun C.J., Chen M.W., Zhou H.S. (2018). Reversible anionic redox activity in Na_3_RuO_4_ cathodes: a prototype Na-rich layered oxide. Energy Environ. Sci..

[22] Balabajew M., Reinhardt H., Bock N., Duchardt M., Kachel S., Hampp N., Roling B. (2016). In-situ Raman study of the intercalation of bis(trifluoromethylsulfonyl)imide ions into graphite inside a dual-ion cell. Electrochim. Acta.

[23] Sole C., Drewet N.E., Hardwick L.J. (2014). In situ Raman study of lithium-ion intercalation into microcrystalline graphite. Faraday Discuss..

[24] Gupta S., Hughes M., Windle A.H., Robertson J. (2014). Charge transfer in carbon nanotube actuators investigated using in situ Raman spectroscopy. J. Appl. Phys..

[25] Dokko K., Shi Q.F., Stefan I.C., Scherson D.A. (2003). In situ Raman spectroscopy of single microparticle Li^+^-intercalation electrodes. J. Phys. Chem. B.

[26] Vecera P., Torres J.C.C., Pichler T., Reich S., Soni H.R., Görling A., Edelthalhammer K., Peterlik H., Hauke F., Hirsch Andreas (2017). Precise determination of graphene functionalization by in situ Raman spectroscopy. Nat. Commun..

[27] Xu H.Y., Bai T.W., Chen H., Guo F., Xi J.B., Huang T.Q., Cai S.Y., Chu X.Y., Ling J., Gao W.W., Xu Z., Gao C. (2019). Low-cost AlCl_3_/Et_3_NHCl electrolyte for high-performance aluminum-ion battery. Energy Storage Mater..

[28] Song Y., Jiao S.Q., Tu J.G., Wang J.X., Liu Y.J., Jiao H.D., Mao X.H., Guo Z.C., Fray D.J. (2017). A long-life rechargeable Al ion battery based on molten salts. J. Mater. Chem. A.

[29] Childress A.S., Parajuli P., Zhu J.Y., Podila R., Rao A.M. (2017). A Raman spectroscopic study of graphene cathodes in high-performance aluminum ion batteries. Nano Energy.

[30] Wang S., Kravchyk K.V., Filippin A.N., Müller U., Tiwari A.N., Buecheler S., Bodnarchuk M.I., Kovalenko M.V. (2018). Aluminum chloride-graphite batteries with flexible current collectors prepared from earth-abundant elements. Adv. Sci..

[31] Alazmi A., Rasul S., Patole S.P., Costa P.M.F.J. (2016). Comparative study of synthesis and reduction methods for graphene oxide. Polyhedron.

